# Neurophysiological features in women with antenatal depressive symptoms: a resting-state quantitative electroencephalography study

**DOI:** 10.1007/s00406-025-02078-w

**Published:** 2025-08-11

**Authors:** Hyeon Ji Kim, Daseul Lee, Jinuk Kim, Na Young Kim, Subeen Hong, Woojae Myung, Hyukjun Lee, Jee Yoon Park

**Affiliations:** 1 Department of Obstetrics and Gynecology, Seoul National University Bundang Hospital, Seongnam, 13619 Republic of Korea; 2 Department of Obstetrics and Gynecology, Seoul National University College of Medicine, Seoul, 03080 Republic of Korea; 3 Department of Neuropsychiatry, Seoul National University Bundang Hospital, Seongnam, 13619 Republic of Korea; 4 Center for Neuroscience Imaging Research, Institute for Basic Science (IBS), Sungkyunkwan University, Suwon, 16499 Republic of Korea; 5 Department of Obstetrics and Gynecology, Seoul St. Mary’s Hospital, College of Medicine, The Catholic University of Korea, Seoul, 06591 Republic of Korea; 6 Department of Psychiatry, Seoul National University College of Medicine, Seoul, 03080 Republic of Korea

**Keywords:** Antenatal depression, Depressive symptoms, Pregnancy, Quantitative electroencephalography (qEEG)

## Abstract

**Background:**

Antenatal depression, which is prevalent during pregnancy, frequently continues into the postpartum period. We aimed to investigate the potential neurophysiological brain changes in women exhibiting antenatal depressive symptoms, using resting-state quantitative electroencephalography (qEEG) patterns as an objective indicator.

**Methods:**

Pregnant women with high-risk conditions were included and evaluated for antenatal depressive symptoms using the Edinburgh Postnatal Depression Scale (EPDS), then divided into groups based on an EPDS score of 10. Resting-state qEEG recordings were then obtained to assess relative power topography within classical frequency bands, comparing these measures across the two groups and examining their correlation with EPDS scores.

**Results:**

Among 36 participants, 12 scored ≥ 10 on the EPDS, indicative of significant depressive symptoms, while 24 scored < 10. Those with scores ≥ 10 exhibited heightened beta power in frontal areas (Fz and F4; *p* < 0.05), along with significant alpha and theta band asymmetry at the T3/T4 (*r* = 0.383, *p* = 0.021) and P3/P4 (*r* = 0.369, *p* = 0.027) sites respectively, positively correlating with EPDS scores.

**Limitations:**

Depressive symptoms were solely evaluated according to the EPDS, which is a screening tool. Additional limitations include the cross-sectional study design and the relatively small sample size, necessitating cautious interpretation of the results.

**Conclusion:**

The distinct qEEG patterns observed in women with EPDS scores ≥ 10 highlight the potential of qEEG as an objective indicator for assessing antenatal depression.

## Introduction

Three to six% of women reportedly experience a major depressive episode during pregnancy or in the weeks or months after giving birth [1]. Prevalence rates ranging from 3.0 to 22.6% have been reported for maternal depression during pregnancy [2–4]. 50% of “postpartum” major depressive episodes actually begin before childbirth [1]. According to the Diagnostic and Statistical Manual of Mental Disorders, Fifth Edition (DSM-5-TR), the specifiers for depressive disorders state that mood episodes can occur during pregnancy or postpartum with a peripartum onset [1]. Thus, depressive symptoms associated with pregnancy have a distinct clinical position, which differentiates them from other forms of depression.

Antenatal depression is associated with adverse perinatal outcomes that may predict postnatal depression [5]. Perceived stress and adverse life events during pregnancy may play an important role in the onset of antenatal depression, with other negative influences including a previous history of depression, low self-esteem, anxiety, low social support, and negative cognitive style [6–10]. Although antenatal depression is relatively under-researched compared to postnatal depression, several studies have found antenatal depression to be equally or more frequent than postpartum depression [6, 11, 12].

Quantitative electroencephalography (qEEG) is widely used across various neuropsychiatric disorders—not only to assess physiological changes in neurodegenerative conditions like Alzheimer’s disease and to evaluate treatment effects in ADHD, but also as a useful tool for elucidating the brain regional mechanisms underlying affective disorders and for investigating neurophysiological indices of depression risk [13–16]. Determining qEEG biomarkers for depression could enhance the detection of risk for the disorder and contribute to the development of biologically driven diagnostic and therapeutic approaches [14, 17, 18]. Patients with depressive episodes have demonstrated elevated alpha and beta waves compared with controls in resting (eyes closed) qEEG recordings. Moreover, increased delta and theta band activity have also been reported [13, 19–21]. For beta power, male outpatients with depression had a higher overall relative beta power than controls [13]. Frontal asymmetry, where higher scores reflect lower left alpha power, has led to greater left cortical activation compared to the right cortex [22].

The majority of previous studies have included general adults experiencing depressive symptoms when not pregnant; thus, little is known about the qEEG findings in women experiencing depressive symptoms during pregnancy. Performing qEEG measurements in pregnant women presents additional challenges compared to that in the general adult population. Data collection, particularly in high-risk maternal patient groups, further exacerbates these challenges. As qEEG signals are closely related to brain activity and emotional state, knowledge of qEEG correlates contributes to interpreting the association between brain functions and antenatal depression [23]. This study aimed to (1) evaluate and compare the qEEG patterns between high-risk pregnant women with high depressive symptoms and those with low depressive symptoms and (2) determine qEEG findings characteristic of antenatal depression based on the results of this comparison.

## Materials and methods

### Participants

This prospective cohort study included pregnant women in their second or third trimesters (> 24 weeks of gestation) who were admitted to the Seoul National University Bundang Hospital, a tertiary referral center for high-risk pregnancies, between April 2020 and May 2021. The study population consisted of women who presented with high-risk conditions, including threatened preterm delivery (preterm labor, preterm premature rupture of membranes), placental previa, fetal growth restriction, or gestational hypertensive disorders. The type of admission was based on the severity of the maternal and fetal condition, with some women being admitted to the general maternity ward, while others requiring admission to a specialized maternal-fetal intensive care unit (MFICU) for more intensive medical care and fetal surveillance. Pregnant women with a history of diagnosed psychiatric disorders or those taking psychotropic medications before or during pregnancy were excluded from the study to ensure antenatal depressive symptoms as the primary focus of investigation. In addition, participants who exhibited signs of delirium, psychosis, or panic-level anxiety requiring psychiatric intervention were not eligible for inclusion. All data collection was performed after clinical stabilization and upon obtaining written informed consent from participants who were alert, oriented, and cognitively capable of cooperating with the procedures. The study was conducted according to the ethical guidelines and approval from the institutional review board (B-1904-537-305).

### Measurements

#### Edinburgh postnatal depression scale

The Edinburgh Postnatal Depression Scale (EPDS) is a self-reported questionnaire for assessing postpartum depression [24]. It consists of 10 items rated on a 4-point scale (0–3), where a higher score indicates greater depression severity, with scores of ≥ 10 considered high risk [25]. Although initially designed for postpartum screening, the EPDS has been widely validated for use during pregnancy due to its emphasis on cognitive and emotional symptoms rather than somatic symptoms that commonly overlap with normal pregnancy-related physiological changes [25–28]. A recent large-scale meta-analysis has demonstrated the EPDS’s strong diagnostic accuracy in antenatal populations, with scores significantly correlating (*r* = 0.59) with structured clinical interviews for major depressive disorder [27, 29]. Given practical constraints in administering extensive clinician-rated assessments to hospitalized pregnant women with high-risk pregnancies, we chose the EPDS for its brevity, practicality, and proven reliability. Participants in our study were classified into two groups based on EPDS scores: those scoring ≥ 10 were considered to have antenatal depressive symptoms, while those scoring < 10 were classified as not exhibiting significant depressive symptoms. Additionally, the Korean version of EPDS (EPDS-K) demonstrates strong psychometric properties, including high internal consistency (Cronbach’s α = 0.84–0.87) and split-half reliability (0.85), further validating its suitability for our study population [30–34].

#### Quantitative electroencephalography

Participants underwent EEG measurements in a quiet, dimly lit room designed to minimize environmental distractions. They were instructed to sit comfortably while the EEG was performed; they kept their eyes closed for 5 min until clear data were recorded. EEG was recorded from 19 Ag/AgCL surface electrodes using EEG cap utilizing an extended international 10/20–location system and a 19-channel wireless EEG acquisition system (MINDD SCAN, Ybrain Inc, Korea). Signals were sampled at 1 kHz and digitized with 24-bit resolution using onboard analog-to-digital converters. Reference electrodes were linked to the ears.

The EEG data were processed and analyzed using the open-source software EEGLAB (v2021.1) implemented in MATLAB^®^ 2021b (MathWorks^,^ Inc., MA, USA) [35]. First, the baseline signal was removed from the raw data measured in the EEG test. The average referencing was established based on the average of the EEG signal using the average reference technique. As all the measurements were conducted in South Korea, a 60 Hz notch filter was applied to remove power line noise. Filtering was performed using finite impulse response (FIR) bandpass filters with a cut-off frequency range of 1–40 Hz, targeting classical EEG sub-bands. The artifact subspace reconstruction (ASR) algorithm was first applied to correct high-amplitude transient artifacts and eliminate bad epochs based on statistical thresholds [36, 37]. Next, the EEG signal was decomposed into 19 independent components using independent component analysis (ICA). To further remove ocular and muscular artifacts, we employed the Multiple Artifact Rejection Algorithm (MARA), which automatically classifies and excludes components associated with electrooculogram (EOG) and electromyogram (EMG) activity [38].

Relative spectral power is used for the accurate analysis of EEG signal data. For spectral analysis, EEG data were segmented into 2-second epochs with 50% overlap and processed using Welch’s method with a Hamming window [39]. EEG spectral power ($\:\mu\:{V}^{2}$) data were averaged into the following 6 bandwidths: delta (1–4 Hz), theta (4–8 Hz), alpha (8–12 Hz), beta (12–25 Hz), high beta (25–30 Hz), and gamma (30–40 Hz). The relative band power was obtained by dividing the total sum of bands for each channel by absolute band power. After preprocessing, intra-hemispheric asymmetry was also computed to assess the balance of neural activity between symmetrical regions of the left and right hemispheres, which was achieved by calculating the relative power difference between homologous electrode pairs (e.g., F3–F4, T3–T4, P3–P4). The asymmetry index (AI) was computed using the following formula:

$$\:AI=\:\frac{{Power}_{left}-{Power}_{right}}{{Power}_{left}+{Power}_{right}}$$

Positive AI values indicate greater activity in the left hemisphere, while negative values suggest greater activity in the right hemisphere. This analysis was performed for each of the six bandwidths (delta, theta, alpha, beta, high beta, and gamma).

This study aimed to investigate the correlation between patients’ clinical scale scores and quantified EEG, utilizing the relative power values from the 19-channel whole brain measurements [40]. All the investigators performing the EEG data review, transformation, and analyses were blinded to the clinical scale scores.

### Statistical analysis

Data were statistically analyzed using IBM SPSS 25.0 (SPSS Inc., Chicago, IL, USA). The values are expressed as mean and standard deviation (mean ± SD) was calculated. Data normality and homogeneity of all the variances were confirmed using Shapiro–Wilk and Levene’s tests, respectively. Differences between the groups in the demographic characteristics, clinical scales, and qEEG data parameters, such as relative power and inter-hemispheric-asymmetry index, were investigated using independent t-tests or Mann–Whitney U tests, depending on the normality of the data distribution. The relationship between the clinical scale scores and z-score relative power was assessed using Pearson’s correlation coefficients. For all the analyses, the significance level was set at *p* = 0.05 in the two-tailed test.

## Results

### Clinical and demographic characteristics

This study included a total of 36 pregnant participants, with 12 women exhibiting EPDS scores ≥ 10 or higher, and 24 women with EPDS scores < 10. In Table 1, various baseline obstetric factors, including maternal age, parity, twin pregnancy status, and the use of assisted reproductive technology, were found to be comparable between the two groups. Several clinical characteristics are known to potentially influence maternal mood during pregnancy, such as the presence of fetal anomalies, employment status, marital status (or the presence of a supportive partner), pre-pregnancy smoking history, smoking during pregnancy, and continued alcohol consumption during pregnancy, yielded no significant differences between the two groups. Women with EPDS scores ≥ 10 were admitted and assessed at an earlier gestational age compared to those with lower scores (median ± SD, 29.5 ± 5.2 vs. 33.8 ± 4.2 weeks; *p* = 0.009, Mann–Whitney test). A significantly higher rate of admission to MFICU was noted in the EPDS scores ≥ 10 group compared to the EPDS scores < 10 groups (100% vs. 54.2%; *p* = 0.006, Chi-square test). No significant differences in the reasons for admissions and the length of hospital stay were observed between the groups.

**Table 1:** Baseline characteristics of the study population according to EPDS scores

	EPDS < 10 (*n* = 24)	EPDS ≥ 10 (*n* = 12)	*P* value
Maternal age (years)	34.83 ± 4.39	33.42 ± 3.55	0.354
Nulliparity	29.2% (7/24)	25.0% (3/12)	> 0.999
Twin pregnancy	37.5% (9/24)	8.3% (1/12)	0.115
Gestational age at assessment (weeks)	33.81 ± 4.24	29.54 ± 5.20	**0.009**
Pre-pregnancy body mass index (kg/m^2^)	22.04 ± 3.40	20.94 ± 3.92	0.240
MFICU admission	54.2% (13/24)	100% (12/12)	**0.006**
Admission duration	11.46 ± 7.74 (13/24)	12.67 ± 7.76	0.623
Fetal anomaly	4.2% (1/24)	8.3% (1/12)	> 0.999
ART	41.7% (10/24)	25.0% (3/12)	0.468
Working at admission / Employment status	66.7% (16/24)	66.7% (8/12)	> 0.999
Marital status / Supportive partner	100.0% (24/24)	100.0% (12/12)	> 0.999
Pre-pregnancy smoking	0.0% (0/24)	8.3% (1/12)	0.333
Smoking during pregnancy	0.0% (0/24)	0.0% (0/12)	> 0.999
Alcohol consumption during pregnancy	0.0% (0/24)	0.0% (0/12)	> 0.999
Maternal underlying disease (indication of admission)			
Hypertensive disorder	16.7% (4/24)	25.0% (3/12)	0.664
Diabetes	12.5% (3/24)	16.7% (2/12)	> 0.999
PTL	33.3% (8/24)	41.7% (5/12)	0.720
PPROM	20.8% (5/24)	33.3% (4/12)	0.443
Cerclage operation	0% (0/24)	8.3% (1/12)	0.333
Placenta previa	16.7% (4/24)	16.7% (2/12)	> 0.999
FGR	8.3% (2/24)	25.0% (3/12)	0.307
Cesarean section	83.3% (20/24)	75.0% (9/12)	0.664
Gestational age at delivery (weeks)	35.83 ± 2.64	34.44 ± 3.05	0.093
Preterm birth < 37 weeks	50.0% (12/24)	66.7% (8/12)	0.481
Preterm birth < 34 weeks	12.5% (3/24)	33.3% (4/12)	0.190

Values are given as Mean and standard deviation (mean ± SD) or percentage (n/N). Values in bold indicate statistical significance (p < 0.05).

*EPDS* Edinburgh Postnatal Depression Scale, *MFICU* maternal-fetal intensive care unit, *ART* assisted reproductive technology, *PTL* preterm labor, *PPROM* preterm premature rupture of membranes, *FGR* fetal growth restriction

### Group differences in the qEEG relative power and inter-hemispheric asymmetry

**Fig. 1:**
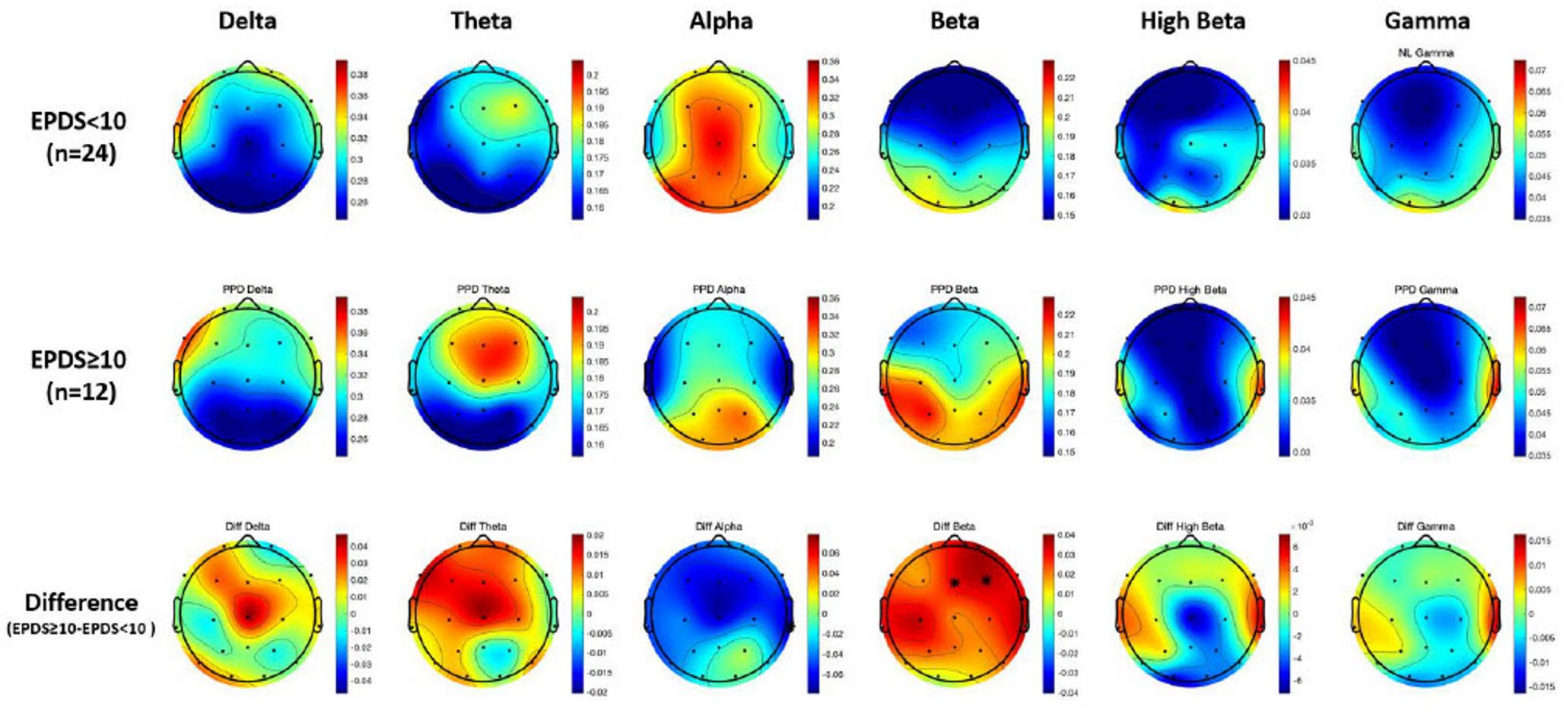
Topographical maps illustrating quantitative electroencephalography (qEEG) power differences across delta, theta, alpha, beta, high-beta, and gamma frequency bands between the women with Edinburgh Postnatal Depression Scale (EPDS) scores ≥ 10 versus women with EPDS scores < 10 groups

**Fig. 2:**
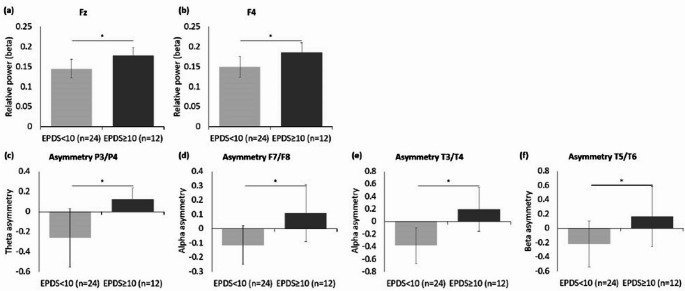
The group differences in the quantitative electroencephalography (qEEG) relative power and inter-hemispheric asymmetry between women with Edinburgh Postnatal Depression Scale (EPDS) scores ≥ 10 and those with EPDS scores < 10, specifically in: **a** beta frequency at Fz, **b** beta frequency at F4, **c** theta asymmetry at P3/P4, **d** alpha asymmetry at F7/F8, **e** alpha asymmetry at T3/T4, and **f** beta asymmetry at T5/T6

Figure 1 showcases topographical maps that highlight the qEEG power differences between the women with EPDS scores ≥ 10 and women with EPDS scores < 10 groups, across the delta, theta, alpha, beta, high beta, and gamma frequency bands. In the theta band, women with EPDS scores ≥ 10 displayed a pronounced increase in power, particularly in the frontal (including prefrontal) and central regions, compared to their counterparts with EPDS scores < 10. On the contrary, in the alpha band, women with EPDS scores ≥ 10 showed a marked reduction in power, predominantly in the temporal and frontal regions. The beta band revealed significant differences, especially at the Fz and F4 channels; women with EPDS scores ≥ 10 had considerably higher beta power (Fig. 2a and b; Fz: *Z* = −1.98, *p* = 0.049; F4: *Z* = −2.18, *p* = 0.029).

Considering inter-hemispheric asymmetry, distinct differences were observed between the groups across several frequency bands. In the theta band, a significant asymmetry difference was evident between the P3–P4 electrode pairs (Fig. 2c; *Z* = −2.58, *p* = 0.009). Specifically, women with EPDS scores ≥ 10 demonstrated heightened neural activity in the left hemisphere (P3). Considering alpha asymmetry, the F7–F8 (Fig. 2d; *Z* = −2.48, *p* = 0.012) and T3–T4 regions (Fig. 2e; *Z* = −2.51, *p* = 0.011) showed significant differences. Beta asymmetry also demonstrated a noticeable difference in the T5-T6 region (Fig. 2f; *Z* = −2.01, *p* = 0.045). Women with EPDS scores ≥ 10 consistently exhibited greater band power in the left hemisphere and lower band power in the right hemisphere compared to the women with EPDS scores < 10. No significant differences were observed in the inter-hemispheric asymmetry in the other frequency bands.

### Associations between maternal depression (EPDS Scores) and qEEG measures

**Fig. 3:**
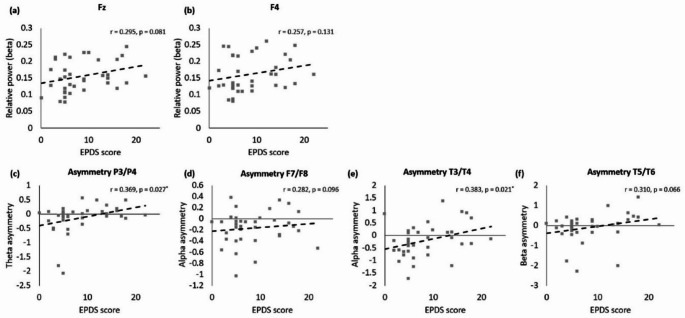
The correlation between the Edinburgh Postnatal Depression Scale (EPDS) scores and quantitative electroencephalography (qEEG) measures for: **a** beta frequency at Fz, **b** beta frequency at F4, **c** theta asymmetry at P3/P4, **d** alpha asymmetry at F7/F8, **e** alpha asymmetry at T3/T4, and **f** beta asymmetry at T5/T6

In the beta band (Fig. 3a, b), although correlations between the EPDS scores and relative power in the Fz and F4 regions were evident, they did not achieve statistical significance. Specifically, the correlation coefficient for the Fz and F4 regions were *r* = 0.295 (*p* = 0.081) and *r* = 0.257 (*p* = 0.131), respectively.

In the theta band (Fig. 3c), a significant positive correlation was observed between the EPDS scores and inter-hemispheric asymmetry between the P3-P4 regions, with a correlation coefficients of *r* = 0.369 (*p* = 0.027). In the alpha band (Fig. 3d, e), the F7–F8 region did not exhibit a significant correlation with the EPDS scores (*r* = 0.282, *p* = 0.106). However, a significant correlation was found between the EPDS scores and inter-hemispheric asymmetry in the T3–T4 regions (*r* = 0.383, *p* = 0.021). In the beta band (Fig. 3f), the T5–T6 inter-hemispheric asymmetry demonstrated a trend towards correlation with the EPDS scores with a correlation coefficient of *r* = 0.310 (*p* = 0.066); however, it was not statistically significant.

## Discussion

We examined the qEEG characteristics in pregnant women with varying degrees of antenatal depressive symptoms in this study. We observed that women exhibiting depressive symptoms demonstrated higher beta power in the frontal areas, specifically within the Fz and F4 regions, than those without depressive symptoms. Additionally, pregnant women with depressive symptoms demonstrated asymmetry in the F7/F8 and T3/T4 sites, with the asymmetry in the T3/T4 area showing a significant correlation with the EPDS scores.

Significant group differences in the qEEG signals, based on the severity of depressive symptoms, were primarily observed in the beta power within the frontal area (Fig. 2a and b). While the majority of previous studies on depressive symptoms focused on the alpha band, our study highlighted the significance of the beta band. Beta band activity is known to correlate with anxiety and ruminating thinking common in patients with depression [17, 41]. An increase in beta band activity usually suggests an over-excited state of the specific brain region [42]. Previous studies have established an association between the increased beta band power and depressive symptoms, particularly highlighting its association with the frontal lobe [43–46]. Our findings were consistent with these findings. Moreover, our study demonstrated antenatal depressive symptoms to be characterized by increased beta band activity within the frontal area, which may be particularly relevant as antenatal depression is often accompanied with anxiety symptoms, with comorbidity rates reaching up to 70%, which are significant risk factors and predictive elements for depressive disorders [22, 47–49].

Our study also revealed various inter-hemispheric asymmetries, notably in the alpha and theta bands, with statistically significant findings in the T3-T4 and P3-P4 regions, respectively (Fig. 2c and e). The alpha asymmetry in qEEG, which has been utilized as an indicator of cortical activity, is associated with emotional processing, mood, psychopathology [50], and depression, thus highlighting its clinical significance [51, 52]. Notably, alpha band asymmetry in our study was observed in the temporal lobe region, which is significantly associated with rumination and depressive disorders [53]. This finding suggests that alpha asymmetry in the temporal region could reflect the characteristics of antenatal depression. The significant correlation between alpha band inter-hemispheric asymmetry in the T3-T4 area and EPDS scores further supports our results (Fig. 3f).

In addition to these findings, our research also highlighted a statistically significant theta band asymmetry in the posterior region (Fig. 2c). Although previous studies have occasionally reported similar observations, the association between depressive symptoms and theta band asymmetry in the posterior area remains relatively underexplored [46, 54]. This gap encourages further research on the role of theta band asymmetry in depression.

Previous qEEG studies on general adults with depression frequently reported an association with increased alpha power, while a review suggested gamma and theta bands as promising indicators [17, 55–57]. In contrast, our study uniquely found increased beta power as an indicator. Additionally, our study found significant correlations between depressive symptoms and inter-hemispheric asymmetry in the alpha and theta bands, specifically within the temporal and parietal regions, respectively. These findings are contradictory to those of previous studies that primarily examined the frontal area for alpha asymmetry and although less common, the frontal and midline areas for theta asymmetry [58–63]. Therefore, our qEEG findings suggest that increased frontal beta power, alpha asymmetry in the temporal area, and theta asymmetry in the parietal area could indicate antenatal depression.

Depression often goes undiagnosed during pregnancy as it is overshadowed by various conditions of physical discomfort, with only approximately 20% of affected women receiving appropriate treatment [64, 65]. Untreated depression can impact infant outcomes, including preterm birth, emotional difficulties, behavior problems, and poor cognitive development [66, 67]. Antenatal depression is also one of the strongest risk factors for postnatal depression, a condition associated with developmental problems in children [67, 68]. Besides these concerning impacts, it is crucial to acknowledge the potential severe consequences of untreated antenatal depression. In extreme cases, this condition can escalate to catastrophic outcomes, such as maternal suicide or infanticide [69]. Hence, timely and accurate diagnosis and treatment of depression during pregnancy are crucial. The findings of this study may aid in the diagnosis of depressive disorders that could be masked by physical discomforts during pregnancy.

### Limitations

The findings of our study should be interpreted with caution in light of certain limitations. First, antenatal depression was defined exclusively by the EPDS, a self-report measure assessing depressive symptoms over the preceding 7 days. This timeframe differs from the 2-week duration required by standard diagnostic criteria for major depressive disorder. Although the EPDS is widely validated for antenatal screening and offers practical benefits for high-risk clinical populations, its shorter reference period may not fully capture the symptom duration and severity defined by formal diagnostic standards. Future studies should consider incorporating clinician-administered scales (e.g., Hamilton Depression Rating Scale or Montgomery–Åsberg Depression Rating Scale) or structured diagnostic interviews to provide more comprehensive evaluations of depression severity and duration. Second, due to the cross-sectional design, distinguishing trait-dependent from state-dependent neurophysiological outcomes remains challenging. Longitudinal studies are required to differentiate stable biomarkers from transient symptom-related changes and to clarify temporal relationships between neurophysiological features and depressive symptoms. Third, pregnant women, particularly the high-risk hospitalized patients included in our study, constitute a vulnerable group due to their physiological status and associated ethical and practical research constraints [70, 71]. Consequently, the relatively small sample size limits the statistical power and generalizability of our results. Future studies with larger and more diverse clinical populations are necessary to substantiate and extend our findings. Fourth, the absence of healthy control participants limits our ability to differentiate qEEG alterations specifically related to antenatal depression from normative pregnancy-related changes. Future research should include healthy comparison groups to clarify these distinctions and to identify neurophysiological markers uniquely associated with antenatal depression. Addressing these limitations through longitudinal designs, multi-dimensional assessments, larger sample sizes, and the inclusion of appropriate control groups would strengthen the robustness and clinical applicability of future research findings.

## Conclusion

Our study evaluated antenatal depressive symptoms in pregnant women using the EPDS and corresponding characteristics of resting-state qEEG. Women with depressive symptoms showed higher beta power in the frontal lobes and asymmetry in the alpha and theta frequency in the temporal and parietal regions, correlating significantly with their EPDS scores. These findings suggest that increased frontal beta activity and temporal alpha asymmetry could be potential markers of antenatal depression, thus highlighting the potential use of qEEG patterns as potential objective measures for identifying antenatal depression.

## Data Availability

The data that support the findings of this study are available from the corresponding author (82839@snubh.org) upon reasonable request.
